# Characteristics and Outcomes of Acute Leukemias in Adolescents and Young Adults with Down Syndrome: A Single-Center Experience

**DOI:** 10.3390/hematolrep17060070

**Published:** 2025-12-18

**Authors:** Marie Nour Karam, Sandra K. Althouse, Madeline G. Andrews, Jenny Chen, Sandeep Batra

**Affiliations:** 1 Department of Pediatrics, Indiana University School of Medicine, Indianapolis, IN 46202, USA; 2 Department of Biostatistics and Data Health Science, Indiana University School of Medicine, Indianapolis, IN 46202, USA; 3 Department of Pediatric Cancer and Blood Disorders, Riley Hospital for Children, Indianapolis, IN 46202, USA; 4 Indiana University Melvin and Bren Simon Comprehensive Cancer Center, Indiana University School of Medicine, Indianapolis, IN 46202, USA

**Keywords:** acute lymphoblastic leukemia, acute myeloid leukemia, Down syndrome, adolescents and young adults

## Abstract

**Background/Objectives:** Acute leukemias in adolescents and young adults (AYAs) with Down Syndrome (DS) are understudied. **Methods**: This was a single-center, retrospective cohort study. Medical records for pediatric DS (*n* = 41) and AYA-DS (*n* = 7) treated with a pediatric chemotherapy regimen for acute leukemia were evaluated. **Results**: Two-year event-free survival (EFS) in AYA DS acute leukemia patients was lower than that in their pediatric DS counterparts (28.6% (Confidence Interval (CI) 4.1, 61.2) vs. 84.9% (CI 69.5, 92.9); *p* = 0.002). **Conclusions**: Additional research is needed to improve outcomes in AYA DS leukemia.

## 1. Introduction

Acute leukemias in adolescents and young adults (AYA), defined as individuals aged 15–39 years, exhibit distinct disease characteristics [1]. A rare and understudied subpopulation within the AYA demographic is the AYA Down Syndrome (DS) cohort with acute leukemias. The risk of de novo acute leukemia is 10–20-fold higher in pediatric (PED) DS than in the general PED population [2]. The incidence of leukemia in the AYA DS population is also increased [3].

Studies have shown that AYA non-DS acute lymphoblastic leukemia (ALL) patients have inferior overall survival (OS) compared to PED non-DS ALL patients [4,5]; however, significant improvements have been made in recent years by prescribing pediatric regimens rather than the typically less rigorous adult regimens used in older adults [5,6]. In contrast, it is unclear whether AYA non-DS patients with myeloid leukemia (ML) benefit from pediatric ML regimens [7]. Interestingly, due to somatic *GATA1* mutations that occur in younger DS patients, outcomes in PED ML-DS (especially those <2 years of age) are superior to those without DS, mainly due to increased chemosensitivity [8].

There is a paucity of data on the characteristics and outcomes of AYA DS ALL and AYA ML-DS [8]. This study aimed to examine the characteristics and treatment outcomes of AYA DS ALL and AYA DS-ML patients treated at our institution.

## 2. Materials and Methods

This was a retrospective, Institutional Review Board-approved cohort study that identified 59 patients with DS who were diagnosed with ALL or ML, treated at Riley Hospital for Children (Indianapolis, IN, USA) between 1987 and 2022, and had a documented medical history of trisomy 21. Of the 59 patients, 11 were excluded due to inadequate information in their electronic medical records (EMR).

The EMR of 48 patients with DS were reviewed, and data were collected on demographic information, prior medical history, laboratory and imaging findings at presentation, treatment regimen, disease course, treatment complications, and treatment outcomes, including relapse and/or death. The patients were stratified into the following categories: PED DS ALL, AYA DS ALL, PED ML-DS, and AYA ML-DS. Patients were allocated to the pediatric group if diagnosed between 0 and 14 years old. Patients were allocated to the AYA group if diagnosed between 15 and 39 years old [1]. Due to the absence of clear differences between myelodysplastic syndrome (MDS) and ML in DS patients [9], patients with MDS were included in the ML cohort (ML-DS). The date of diagnosis for all patients was defined as the day the diagnostic bone marrow biopsy results were available. In infants first diagnosed with transient myeloproliferative disorder (TMD) which eventually advanced into ML, the diagnosis date was based on the date when conversion into ML was confirmed. When appropriate, obesity was defined as a Body Mass Index (BMI) greater than or equal to 30.0 [10].

All patients were treated according to the standard-of-care protocol of the appropriate cooperative group at that time. Treatment-related complications such as hypertension and venous thromboembolic events (VTEs) were defined according to the Common Terminology Criteria for Adverse Events (CTCAEs) version 5.0 [11]. Corrected serum calcium levels were calculated from the total serum calcium levels at diagnosis using the following formula [12]:

Measured Corrected calcium (mg/dL) = 0.8 (4 − serum albumin (g/dL))

Data analysis was conducted using SAS software version 9.4 (SAS Institute Inc., Cary, NC, USA). A *p*-value < 0.05 denoted statistical significance for all tests. Baseline demographic and disease characteristics were summarized as median (range) for continuous variables and number (percent) for categorical variables. Comparisons between groups were performed using Chi-square tests (or Fisher’s Exact test, where appropriate) for categorical variables, or the Wilcoxon rank sum test for continuous variables. The Kaplan–Meier method was used to analyze OS, disease-free survival (DFS), and event-free survival (EFS) using the log-rank test to compare groups, where appropriate. 

If a patient did not die, they were censored on the last date known to be alive. DFS was calculated from the date of diagnosis to relapse or death. In the calculation of EFS, “events” include death, relapse, and VTE occurrence.

No generative Artificial Intelligence (AI) was used in this study.

## 3. Results

The demographic features and key clinical and laboratory findings of patients with DS ALL and ML-DS are outlined in Table 1 and Table 2, and Supplemental Table S1. 

Our cohort consisted of 6 AYA DS ALL, 21 PED DS ALL, 1 AYA ML -DS, and 20 PED ML-DS patients. In the PED DS ALL (*n* = 21) and AYA DS ALL (*n* = 6) groups, the median age at diagnosis was 3 years and 17 years, and the BMI at diagnosis was 17.2 and 32.7 (*p* = 0.036), respectively (Table 1). The blast count, platelet count, white blood cell (WBC) count, hemoglobin, aspartate transaminase (AST), alanine transaminase (ALT), lactate dehydrogenase (LDH), uric acid, alkaline phosphatase, and albumin levels at presentation were similar between the two groups (Table 2; *p* > 0.3). The AYA DS ALL cohort had higher median adjusted serum calcium levels compared to the PED DS ALL cohort, respectively (9.7 mg/dL vs. 9.2 mg/dL; *p* = 0.02, Table 2). Patients with AYA DS ALL had lower total serum phosphorus levels than those with PED DS ALL (4 mg/dL vs. 5.4 mg/dL; *p* = 0.004, Table 2). VTEs occurred more frequently in the AYA DS ALL cohort (*n* = 3 (50%) vs. *n* = 2 (9.5%), *p* = 0.056), though this analysis was not statistically significant. These VTEs occurred during or after induction in two patients, following CAR-T therapy in one patient, and at an indeterminate time in treatment for the final patient due to incomplete, older records. No difference was noted in febrile neutropenia episodes or the incidence of hypertension (Table 2).

**Figure 1:**
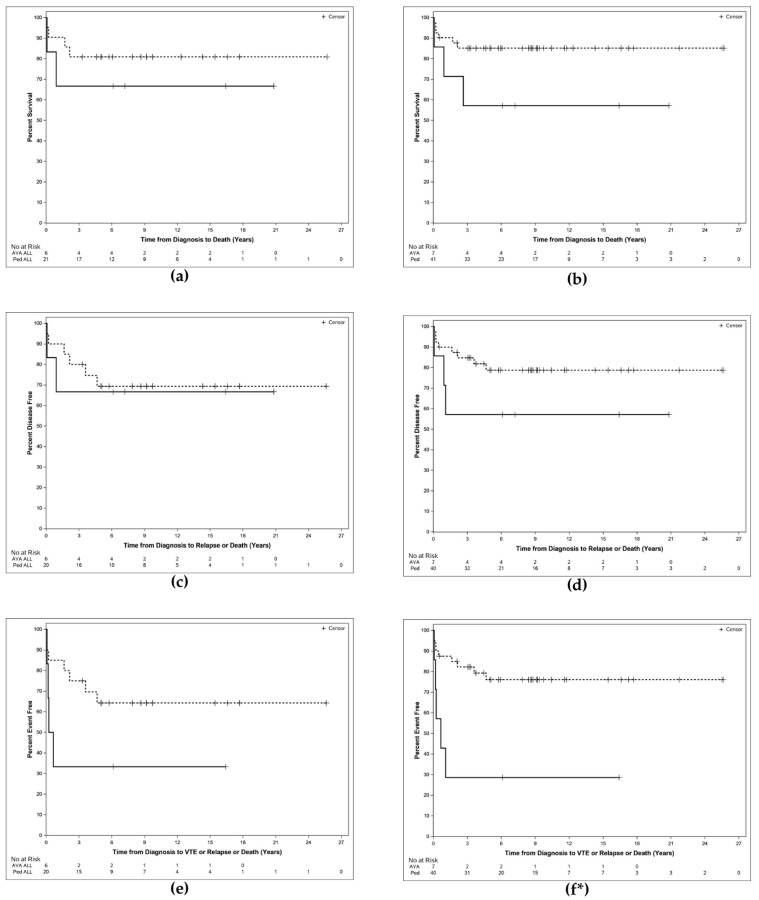
Kaplan–Meier Plots displaying OS, DFS, and EFS. (**a**): OS of PED DS ALL (dashed) vs. AYA DS ALL (solid). (**b**): OS of PED DS (ALL + ML) (dashed) vs. AYA DS (ALL + ML) (solid). (**c**): DFS of PED DS ALL (dashed) vs. AYA DS ALL (solid). (**d**): DFS of PED DS (ALL + ML) (dashed) vs. AYA DS (ALL + ML) (solid). (**e**): EFS of PED DS ALL (dashed) vs. AYA DS ALL (solid). (**f**): EFS of PED DS (ALL + ML) (dashed) vs. AYA DS (ALL + ML) (solid). * = *p* < 0.005. AYA = Adolescents and Young Adults; PED = pediatric; DS = Down Syndrome; ALL = Acute Lymphoblastic Leukemia; ML = myeloid leukemia; OS = Overall Survival; DFS = Disease-Free Survival; EFS = Event-Free Survival.

The PED DS ALL cohort had consistently higher rates of 2-year OS (85.7, CI 62.0–95.2; vs. 66.7, CI 19.5–90.4, Figure 1a), DFS (85.0, CI 60.4, 94.9; vs. 66.7, CI 19.5–90.4, Figure 1c), and EFS (80.0, CI 55.1–92.0; vs. 33.3, CI 4.6–67.6, Figure 1e) than their AYA DS ALL counterparts, but this difference was not statistically significant (*p* = 0.43, *p* = 0.80, *p* = 0.09, respectively) (Table 3, Figure 1).

Comparative analysis between the combined PED (ALL + ML) cohort and AYA (ALL + ML) cohort revealed similar trends, whereby 2-year OS (87.7, CI 73.0–94.7; vs. 71.4, CI 25.8–92.0, Figure 1b), DFS (87.4, CI 72.4–94.6; vs. 57.1, CI 17.2–83.7, Figure 1d), and EFS (84.9, CI 69.5–92.9; vs. 28.6, CI 4.1–61.2, Figure 1f) in the PED cohort were superior. The differences in EFS were statistically significant in this analysis (*p* < 0.002), but the differences in OS and DFS were not (*p* = 0.08, *p* = 0.16, respectively) (Table 3, Figure 1). To confirm these results, the singular AYA ML-DS patient was excluded, and the analysis was repeated. Differences in EFS remained statistically significant in AYA vs. PED. (33.3, CI 4.6–67.6; vs. 84.9, CI 69.5–92.9; *p* = 0.0078).

In the ML-DS cohorts, 6 subjects out of 21 (28.57%) had confirmed M7 subtype, or Acute Megakaryoblast Leukemia (AMKL) subtype of the French American British (FAB) classification, and 2 (9.09%) were of the M0, or Acute Myeloblastic Undifferentiated Phenotype. 12 (57%) PED ML-DS patients were diagnosed in early childhood (age < 2 years), 7 were diagnosed between the ages of 2 and 15 years, and 1 was of unknown age at the time of diagnosis (Supplemental Table S1). No relapses occurred in the PED ML-DS group. 

The single AYA ML-DS patient, aged 26 years at diagnosis, presented with several key presenting features, including hyperleukocytosis (WBC count 137.2 k/cumm). Cytogenetic analyses revealed numerous genetic abnormalities, including *FTL3-TKD*, *GATA2*, *NPM1*, *RNF43*, and *CBL* mutations. The patient had a suboptimal response to induction chemotherapy per AAML0431 protocol, requiring a switch to AAML0131 with midostaurin for Intensification I. Midostaurin was discontinued for Intensification II because of likely midostaurin-induced pulmonary toxicity. The first relapse occurred 8 months after treatment with daunorubicin and cytarabine in cycle 1, followed by FLAG (fludarabine, cytarabine, granulocyte colony-stimulating factor, and idarubicin) and gilteritinib in cycles 2 and 3. Stem cell treatment (SCT) was discussed after remission was achieved, but declined due to malalignment with the goals of care. He ultimately relapsed 9 months later and was treated with oral palliative chemotherapy before he died.

Cytogenetic, genetic, and molecular data are summarized in Supplemental Tables S2–S4. Genetic and cytogenetic analyses did not reveal any major differences in any analysis conducted. The most common genetic or cytogenetic abnormality was a *RUNX1* gain (19.5% of all PED patients, Supplemental Table S3). An *ETV6-RUNX1* fusion was the most common in the ALL cohort and was seen in both the AYA and PED groups (33.3% of AYA DS ALL, 14.3% of PED DS ALL, 10.42% of total cohort; Supplemental Tables S2 and S3) but was not present in any ML patient. *CRLF2* rearrangements (14.3% of PED DS ALL, 7.3% of all PED patients; Supplemental Tables S2 and S3) were also common in the PED DS ALL cohort but absent in the AYA DS ALL and AYA ML-DS cohorts. A total of 5 trisomies were documented in chromosomes 8, 9, or 11 (4 PED ML-DS; 1 PED DS ALL, Supplemental Tables S2 and S4). No trisomy was detected in the AYA population. Of note, the only patient with documented Ph-like ALL had numerous genetic aberrations, including *JAK* mutations, a *CRLF2* rearrangement, a *P2RY8-CRLF2* fusion, an *IKZF1-PAX5* fusion, and hyperdiploidy. One patient (PED DS ALL) had a documented loss of *MLL*, while another patient (PED ML-DS) had a documented gain of *MLL*.

## 4. Discussion

In this retrospective study, we compared the outcomes of PED DS and AYA DS patients with acute leukemia.

Our results showed that patients with AYA DS ALL had higher BMIs at presentation than PED DS ALL. Both AYA DS and PED DS are at an increased risk of being overweight or obese. It is also known that the risk of obesity tends to increase with age throughout adolescence and adulthood [13]. In both PED and AYA non-DS ALL populations, obese and/or overweight pediatric patients tend to demonstrate worse EFS [13,14] and increased relapse [15]. One possible mechanism is adipocyte modulation of the leukemic microenvironment, causing decreased chemotherapy cytotoxicity [5,16]. Leukemic cells have also been shown to migrate into adipocytes, thereby shielding them from the effects of chemotherapy [5,17]. Further research is required to delineate the relationship between obesity and outcomes in patients with DS AYA ALL.

In our cohort of patients, treatment-related toxicities such as febrile neutropenia and hypertension were similar between PED and AYA DS ALL patients. Half of our AYA DS ALL patients (3/6, 50%) experienced a VTE, which is high, but the number of patients in our study was small. Previous research indicated that VTEs occur frequently in the AYA population with leukemias, with one study showing a VTE rate of 21% and another showing a VTE rate of 4.76% [18,19]. Moreover, the VTE occurrence rate is greater in AYA cohorts than their pediatric counterparts and more frequent in obese patients [18,20]. Given these observations, the potential utility of prophylactic anticoagulation in AYA leukemia has been investigated. One retrospective study of VTEs in AYA patients showed that Low-molecular-weight heparin (LMWH) did not decrease VTE incidents in the overall cohort, nor was it proven effective in obese patients after subgroup analysis [20]. However, in the PREVAPIX-ALL trial of pediatric leukemias, VTE prophylaxis significantly mitigated the number of VTEs in obese patients only [21,22]. These findings need to be further studied in larger patient cohorts, including the possible association between VTEs and obesity, and specifically in AYA DS patients.

At diagnosis, AYA DS ALL patients had higher serum calcium levels but lower phosphorus levels than PED DS ALL patients. Phosphorus levels in children are typically higher than in adults [23] (normal range is approximately 4.5–6.5 mg/dL in children, 2.5–4.5 mg/dL in adults [24]), which could explain our results. When accounting for age-related differences, children tend to have higher baseline total calcium levels due to their physiologic growth needs [25]; however, none of the patients in our ALL group met the criteria for either hypercalcemia or hypocalcemia at diagnosis, and albumin levels were not significantly different (Table 2).

In our study, all patients with AYA DS ALL were treated with a pediatric regimen, yielding excellent OS and DFS (Figure 1 and Supplemental Figure S1) similar to previously published studies [5,6]. These results support the use of pediatric regimens in AYA DS ALL patients. The OS, EFS, and DFS were higher in the PED DS ALL group (Figure 1, Table 3) than in the AYA DS ALL group; however, these metrics did not achieve statistical significance. Due to the rarity of DS ALL in the AYA population, we identified only 6 patients; thus, our analyses were likely underpowered to draw major conclusions. Interestingly, when we grouped the PED (ALL + ML) cohort and compared the outcomes to the AYA (ALL + ML) cohort, the PED group had improved outcomes, and the differences in EFS were statistically significant (Figure 1b,d,f; *p* = 0.0021), suggesting that overall, AYA DS leukemia patients had inferior outcomes compared to their pediatric counterparts. This is possibly due to differences in the occurrence of adverse events, particularly VTEs, as the only variable that differentiates DFS from EFS is VTE occurrence, and only EFS achieved statistical significance. Our patient cohort and analyses did not include comparisons between AYA DS ALL and AYA non-DS ALL. However, comparisons between PED DS ALL and PED non-DS ALL in other studies showed decreased survival owing to a combination of increased treatment-related mortality (TRM) and worse relapse rates in the PED DS ALL group [8].

To improve outcomes in AYA DS patients, and to continue with the principle of applying pediatric regimens to AYA ALL, novel treatments introduced in the pediatric population should be studied and incorporated into AYA DS regimens [26]. For example, AYA DS patients may benefit from immunotherapies such as blinatumomab, which is now a standard treatment component of the Children’s Oncology Group (COG) regimen in B-ALL [27].

Our study presented limitations, including a small sample size and a retrospective study design. In conclusion, further large cooperative, multi-center studies are needed to report the outcomes of AYA DS patients with acute leukemia, study the impact of obesity, and evaluate risk factors associated with VTE in this rare AYA population. Efforts should be made to improve outcomes in this subgroup through a combination of immunotherapy and chemotherapy, as is being performed in the pediatric population.

## Figures and Tables

**Table 1:** Demographic characteristics of the AYA DS ALL and PED DS ALL cohorts.

Demographic Characteristic		Overall *n* = 27	PED DS ALL *n* = 21	AYA DS ALL *n* = 6	*p* Value	
Age at Diagnosis		4 (2–20)	3 (2–12)	17.0 (15.0–20.0)	<0.001 *	
Sex	F	11 (40.7%)	8 (38.1%)	3 (50.0%)	0.6	
	M	16 (59.3%)	13 (61.9%)	3 (50.0%)		
Race	Asian	1 (4.5%)	1 (5.6%)	0 (0.00%)	1.0	
	African American	1 (4.5%)	1 (5.6%)	0 (0.00%)		
	White	20 (90.9%)	16 (88.9%)	4 (100.0%)		
Ethnicity	Hispanic/Latino	2 (8.7%)	2 (10.5%)	0 (0.00%)	1.0	
	Not Hispanic/Latino	21 (91.3%)	17 (89.5%)	4 (100.0%)		
BMI group	Underweight	8 (29.6%)	8 (38.1%)	0 (0.00%)	0.06	
	Normal	3 (11.1%)	3 (14.3%)	0 (0.00%)		
	Overweight	2 (7.4%)	1 (4.8%)	1 (16.7%)		
	Obese	1 (3.7%)	0 (0.00%)	1 (16.7%)		
	Unknown	13 (48.1%)	9 (42.9%)	4 (66.7%)		
Risk stratification	Standard risk	6 (46.2%)	6 (66.7%)	0 (0.00%)	0.09	
	High risk	6 (46.2%)	3 (33.3%)	3 (75.0%)		
	Very high risk	1 (7.7%)	0 (0.00%)	1 (25.0%)		
CNS Status	CNS1	23 (88.5%)	18 (90.0%)	5 (83.3%)	0.5	
	CNS2	2 (7.7%)	1 (5.0%)	1 (16.7%)		
	CNS2B	1 (3.8%)	1 (5.0%)	0 (0.00%)		
BMI at diagnosis		17.4 (14.4–35.5)	17.2 (14.4–27.2)	32.7 (29.8–35.5)	0.036 *	

Note: Values expressed as n (%), or median (range). All lab values were obtained at the time of diagnosis. *p*-value comparisons across group categories are based on the chi-square test (or Fisher’s Exact test) for categorical variables; *p*-values for continuous variables are based on the Wilcoxon Rank Sum. * = statistically significant (*p* < 0.05). AYA = Adolescents and Young Adults; PED = pediatric; ALL = Acute Lymphoblastic Leukemia; DS = Down Syndrome; BMI = Body-Mass Index; CNS = Central Nervous System.

**Table 2:** Diagnostic and post-treatment characteristics of the AYA DS ALL and PED DS ALL cohorts.

Lab Value at Diagnosis	Overall *n* = 27	PED DS ALL *n* = 21	AYA DS ALL *n* = 6	*p* Value
ANC (/mm^3^)	0.7 (0.1–8.7)	0.8 (0.1–8.7)	0.2 (0.1–4.8)	0.7
% Blasts Peripheral Smear (/mm^3^)	67.5 (0–97)	69.5 (0–97)	56.0 (19.0–92.0)	0.4
WBC (/mm^3^)	9.7 (2.2–366.5)	13.6 (2.17–241.6)	5.4 (5.2–366.5)	0.4
Hemoglobin (g/dL)	8.6 (4–13)	8.6 (4–12.6)	8.4 (6.2–13.0)	0.8
Platelet (/mm^3^)	40 (5–131)	41.0 (5.0–131.0)	39.0 (6.0–131.0)	1.0
LDH (U/L)	670 (209–2612)	771.5 (209–1700)	625.0 (229.0–2612.0)	0.8
Uric Acid (mg/dL)	6.3 (3.4–10.1)	6.4 (3.4–10.1)	6.3 (6.2–8.1)	0.6
Calcium (mg/dL)	9.2 (8.7–10.2)	9.3 (8.7–10.2)	9 (8.7–9.1)	0.02 *
Adjusted Calcium (mg/dL)	9.6 (8.9–10.4)	9.2 (8.9–9.6)	9.7 (9.1–10.4)	0.02 *
Phosphorous (mg/dL)	5.3 (3.4–6.8)	5.4 (4.5–6.8)	4 (3.4–4.6)	0.004 *
Albumin (g/dL)	3.5 (2.7–4.2)	3.5 (2.7–4.2)	3.5 (2.9–4.1)	0.8
Alkaline Phosphatase (U/L)	129 (63–304)	133.0 (63.0–304.0)	102.0 (65.0–198.0)	0.4
BUN (mg/dL)	16 (5–26)	14.5 (6–26)	16.0 (5.0–17.0)	0.8
ALT (U/L)	25.5 (4–450)	25.5 (4–100)	40.5 (13–450)	0.3
AST (U/L)	46 (8–291)	40.5 (8–131)	57.0 (19.0–291.0)	0.4
HTN, induction	7 (28.0%)	6 (31.6%)	1 (16.7%)	1.0
VTE Occurrence	5 (18.5%)	2 (9.5%)	3 (50.0%)	0.056
Febrile Neutropenia, Induction	24 (88.9%)	18 (85.7%)	6 (100.0%)	1.0

Note: Values expressed as n (%), or median (range). All lab values were obtained at diagnosis. *p*-value comparisons across group categories are based on the chi-square test (or Fisher’s Exact test) for categorical variables; *p*-values for continuous variables are based on the Wilcoxon Rank Sum. While complications of hypertension and febrile neutropenia were only considered if they happened during induction, venous thromboembolic events were considered at any phase of treatment. * = statistically significant (*p* < 0.05). AYA = Adolescents and Young Adults; PED = pediatric; ALL = Acute Lymphoblastic Leukemia; DS = Down Syndrome. ANC = Absolute Neutrophil Count; WBC = White Blood Cells; LDH = Lactate Dehydrogenase; BUN = Blood Urea Nitrogen; ALT = Alanine Aminotransferase; AST = Aspartate Aminotransferase; HTN = Hypertension; VTE = venous thromboembolism.

**Table 3:** Summary of OS, DFS, and EFS for PED DS ALL, AYA DS ALL, PED DS (ALL + ML), and AYA DS (ALL + ML).

Outcome	PED DS ALL	AYA DS ALL	Log Rank *p*-Value	PED DS (ALL + AML)	AYA DS (ALL + AML)	Log Rank *p*-Value
Overall Survival (OS) %						
Median (years)	NR	NR	0.4261	NR	NR	0.08
1-year probability	90.5	66.7		90.2	71.4	
2-year probability	85.7	66.7		87.7	71.4	
Disease Free Survival (DFS) %						
Median (years)	NR	NR	0.7963	NR	NR	0.16
1-year probability	90.0	66.7		90.0	71.4	
2-year probability	85.0	66.7		87.4	57.1	
Event Free Survival (EFS) %						
Median (years)	NR	0.4	0.0954	NR	0.6	0.002
1-year probability	85.0	33.3		87.5	42.9	
2-year probability	80.0	33.3		84.9	28.6	

PED DS ALL: *n* = 21 for OS, *N* = 20 for DFS/EFS. AYA DS ALL: *n* = 6. PED DS (ALL + ML): *n* = 41 for OS, N = 40 for DFS/EFS. AYA DS (ALL + ML): *n* = 7. NR = Not Reached. AYA = Adolescents and Young Adults; PED = pediatric; ALL = Acute Lymphoblastic Leukemia; ML = Myeloid Leukemia; DS = Down Syndrome; OS = Overall Survival; DFS = Disease-Free Survival; EFS = Event-Free Survival.

## Data Availability

The data presented in this study are available on request from the corresponding author due to HIPAA regulations restricting access to protected health information.

## Supplementary Materials

The following supporting information can be downloaded at: https://www.mdpi.com/article/10.3390/hematolrep17060070/s1. Figure S1: Kaplan–Meier Plots of OS, DFS, and EFS; Table S1: Demographic, diagnostic, and post-treatment characteristics of the AYA ML-DS and Ped ML-DS cohorts. Table S2: Cytogenetic, genetic, and molecular features in the AYA ALL and PED ALL cohorts. Table S3: Cytogenetic, genetic, and molecular features in the combined Ped and AYA cohorts. Table S4: Cytogenetic, genetic, and molecular features in the PED ML-DS and AYA ML-DS cohorts. Table S5: Summary of OS, DFS, and EFS for PED ML-DS and AYA ML-DS.
