# Supplementary material for: Characteristics and Outcomes of Acute Leukemias in Adolescents and Young Adults with Down Syndrome: A Single-Center Experience
Source: Hematol Rep. 2025 Dec 18;17(6):70. doi: 10.3390/hematolrep17060070 (PMC12733178; doi:10.3390/hematolrep17060070)
Supplement: Supplementary file 1 [file hematolrep-17-00070-s001.zip › hematolrep-3920365-supplementary_17.11.pdf]

Supplemental Table S1. Demographic, diagnostic, and post-treatment characteristics of the AYA ML-DS and Ped ML-DS cohorts.

Variable		All<br>N = 21	Ped ML-DS<br>N = 20	AYA ML-DS<br>N = 1
Age at Diagnosis		1 (0, 26)	1 (0.002739, 3)	26.0 (26.0, 26.0)
Age group	<2 y/o	12 (57.1%)	12 (60.0%)	0 (0%)
	2-15 y/o	7 (33.3%)	7 (35%)	0 (0%)
	15–29 y/o	1 (4.8%)	0 (0%)	1 (100.0%)
	Unknown	1 (4.8%)	1 (5.0%)	0 (0%)
Sex	F	11 (52.4%)	11 (55.0%)	0 (0%)
	M	10 (47.6%)	9 (45.0%)	1 (100.0%)
Race	Asian	2 (10.0%)	2 (10.5%)	0 (0%)
	Black/African American	1 (5.0%)	1 (5.3%)	0 (0%)
	White	17 (85.0%)	16 (84.2%)	1 (100.0%)
Ethnicity	Hispanic/Latino	2 (10.0%)	2 (10.5%)	0 (0%)
	Not Hispanic or Latino	18 (90.0%)	17 (89.5%)	1 (100.0%)
BMI at diagnosis		17.1 (11.1, 31.1)	16.5 (11.12, 30.1)	31.1 (31.1, 31.1)
BMI group	Underweight	7 (33.3%)	7 (35.0%)	0 (0%)
	Healthy	1 (4.8%)	1 (5.0%)	0 (0%)
	Obese	2 (9.5%)	1 (5.0%)	1 (100.0%)
	Unknown	11 (52.4%)	11 (55.0%)	0 (0%)
CNS Status	CNS1	19 (90.5%)	18 (90.0%)	1 (100.0%)
	CNS2	1 (4.8%)	1 (5.0%)	0 (0%)
	CSN1	1 (4.8%)	1 (5.0%)	0 (0%)
ANC/mm^3		1.7 (0.3, 4.3)	1.7 (0.3, 4.3)	3.4 (3.4, 3.4)
Blast %/mm^3		15 (0, 94)	13.5 (0, 94)	94.0 (94.0, 94.0)
WBC/mm^3		6.7 (1.3, 216.8)	6.4 (1.29, 216.8)	137.2 (137.2, 137.2)
Hgb (g/dL)		8.7 (3, 14)	8.8 (3.0, 14.0)	8.2 (8.2, 8.2)
Platelets/mm^3		35 (6, 434)	35.0 (6.0, 434.0)	29.0 (29.0, 29.0)
LDH (U/L)		1019 (204, 4814)	935.0 (204.0, 4814.0)	1313.0 (1313.0, 1313.0)
Uric Acid (mg/dL)		4.9 (2.9, 13.6)	4.8 (2.9, 13.6)	6 (6, 6)
Calcium (mg/dL)		9.2 (7.2, 10.7)	9.2 (7.2, 10.7)	8.2 (8.2, 8.2)
Phosphorous (mg/dL)		4.6 (3.5, 6)	4.8 (3.7, 6)	3.5 (3.5, 3.5)
Albumin (g/dL)		3.4 (1.6, 7)	3.4 (1.6, 7)	3 (3, 3)
Alkaline Phosphatase (U/L)		131 (68, 207)	132.0 (97.0, 207.0)	68.0 (68.0, 68.0)
BUN (mg/dL)		10.5 (5, 85)	11.0 (5.0, 85.0)	10.0 (10.0, 10.0)
ALT (U/L)		26.5 (11, 225)	29.0 (12.0, 225.0)	11.0 (11.0, 11.0)
AST (U/L)		41 (18, 455)	38.0 (18.0, 455.0)	44.0 (44.0, 44.0)
HTN, induction		2 (9.52%)	2 (10.0%)	0 (0%)
Febrile Neutropenia, induction		18 (85.7%)	17 (85.0%)	1 (100.0%)
VTE Events		0 (0%)	0 (0%)	0 (0%)

Note: Values expressed as n (%), or median (range). All lab values were obtained at diagnosis. While complications of hypertension and febrile neutropenia were only considered if they happened during induction, venous thromboembolic events were considered at any phase of treatment. Statistical analyses not conducted due to inadequate sample size. AYA = Adolescents and Young Adults; ML = Myeloid Leukemia; DS = Down Syndrome; ANC = Absolute Neutrophil Count; WBC = White Blood Cells; LDH = Lactate Dehydrogenase; BUN = Blood Urea Nitrogen; ALT = Alanine Aminotransferase; AST = Aspartate Aminotransferase; HTN = Hypertension; VTE = venous thromboembolism.

Supplemental Table S2. Cytogenetic, Genetic, and Molecular Features in the AYA ALL and PED ALL cohorts.

Cytogenetic, Genetic, and Molecular Features	AYA DS ALL (N = 6)	PED DS ALL (N = 21)	Fisher's Exact Test *p*-Value
CRLF2 rearrangement	0(0.0%)	3(14.3%)	1.0000
ETV6-RUNX1 fusion	2(33.3%)	3(14.3%)	0.3031
Gain of *RUNX1*	0(0.0%)	4(19.0%)	0.5453
Hyperdiploidy	0(0.0%)	1(4.8%)	1.0000
IKZF1-PAX5 fusion	0(0.0%)	1(4.8%)	1.0000
JAK mutation	0(0.0%)	1(4.8%)	1.0000
KRAS mutation	1(16.7%)	0(0.0%)	0.2222
Trisomy 9	0(0.0%)	1(4.8%)	1.0000

Values expressed as N (%). Statistical significance was not reached ($p > 0.05$).

Supplemental Table S3. Cytogenetic, Genetic, and Molecular features in the combined PED and AYA cohorts.

Cytogenetic, Genetic, and Molecular Features	AYA (ALL + ML) (N = 7)	PED (ALL + ML) (N = 41)	Fisher's Exact Test *p*-Value
CRLF2 rearrangement	0(0.0%)	3(7.3%)	1.000
ETV6-RUNX1 fusion	2(28.6%)	3(7.3%)	0.1483
GATA1 mutation	1(14.3%)	1(2.4%)	0.2730
Gain of *RUNX1*	0(0.0%)	8(19.5%)	0.5829
IKZF1-PAX5 fusion	0(0.0%)	1(2.4%)	1.0000
JAK mutation	0(0.0%)	4(9.8%)	1.0000
KRAS mutation	1(14.3%)	0(0.0%)	0.1458
Trisomy 11	0(0.0%)	2(4.9%)	1.0000
Trisomy 8	0(0.0%)	3(7.3%)	1.0000
Hyperdiploidy	0(0.0%)	1(2.4%)	1.0000

Values expressed as N (%). Statistical significance was not reached ($p > 0.05$).

Supplemental Table S4. Cytogenetic, Genetic, and Molecular features in the PED ML-DS and AYA ML-DS cohorts.

Cytogenetic, Genetic, and Molecular Features	AYA ML-DS Patients (N = 1)	PED ML-DS Patients (N = 20)
GATA1 mutation	0(0.0%)	1(5.0%)
GATA2 Mutation	1(100.0%)	0(0.0%)
FLT3-IKD Mutation	1(100.0%)	0(0.0%)
NPM1 Mutation	1(100.0%)	0(0.0%)
Gain of *RUNX1*	0(0.0%)	4(20.0%)
JAK mutation	0(0.0%)	3(15.0%)
Trisomy 11	0(0.0%)	1(5.0%)
Trisomy 8	0(0.0%)	3(15.0%)

Values expressed as N (%). Statistical analyses not conducted due to inadequate sample size.

Supplemental Table S5. Summary of OS, DFS, and EFS for PED ML-DS and AYA ML-DS cohorts.

Outcome	Ped ML-DS (N = 20)	AYA ML-DS (N = 1)
**Overall Survival % (CI)**		
**Median in years (95% CI)**	NR (NR, NR)	2.6 (NR, NR)
**1 year probability (95% CI) (%)**	90.0 (65.6, 97.4)	100 (100, 100)
**2 year probability (95% CI) (%)**	90.0 (65.6, 97.4)	100 (100, 100)

**Disease Free Survival % (CI)**		
**Median in years (95% CI)**	NR (NR, NR)	1.1 (NR, NR)
**1 year probability (95% CI) (%)**	90.0 (65.6, 97.4)	100 (100, 100)
**2 year probability (95% CI) (%)**	90.0 (65.6, 97.4)	0 (NR, NR)
**Event Free Survival % (CI)**		
**Median in years (95% CI)**	NR (NR, NR)	1.1 (NR, NR)
**1 year probability (95% CI) (%)**	90.0 (65.6, 97.4)	100 (100, 100)
**2 year probability (95% CI) (%)**	90.0 (65.6, 97.4)	0 (NR, NR)

NR = Not Reached. CI = Confidence Interval. All confidence intervals are 95% confidence intervals. Statistical analyses not conducted due to inadequate sample size.
