# Supplementary material for: Characteristics and Outcomes of Acute Leukemias in Adolescents and Young Adults with Down Syndrome: A Single-Center Experience
Source: Hematol Rep. 2025 Dec 18;17(6):70. doi: 10.3390/hematolrep17060070 (PMC12733178; doi:10.3390/hematolrep17060070)
Supplement: Supplementary file 1 [file hematolrep-17-00070-s001.zip › hematolrep-3920365-to_supplementaryFig1.pdf]

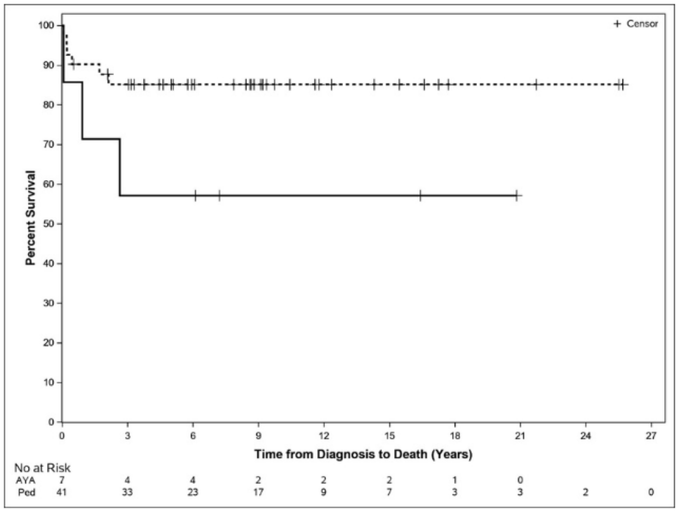


**(a)**

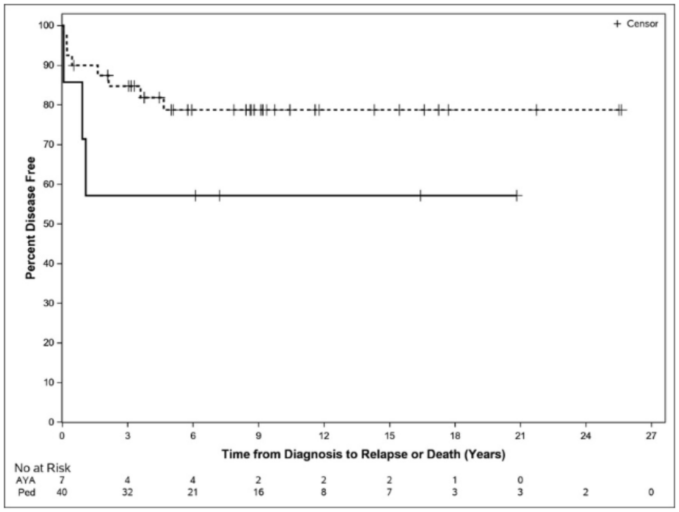


**(b)**

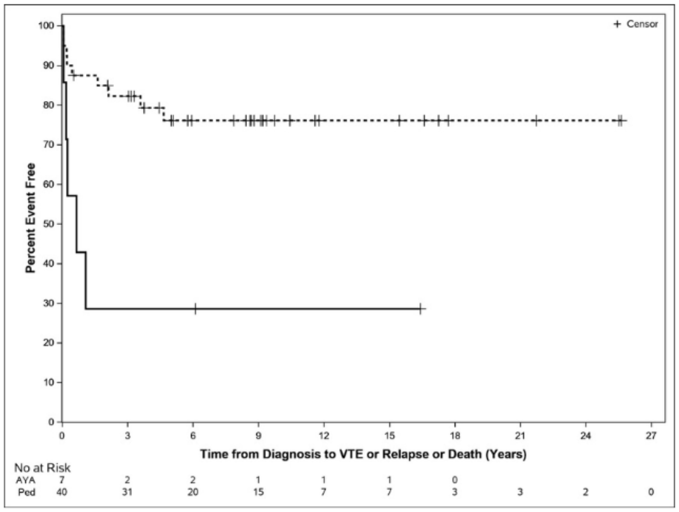


**(c)**

**Supplemental Figure 1: Kaplan-Meier Plots of OS, DFS, and EFS. a:** OS of PED ML-DS (dashed) vs. AYA ML-DS (solid). **b:** DFS of PED ML-DS (dashed) vs. AYA ML-DS (solid). **c:** Event-Free Survival (EFS) of PED ML-DS (dashed) vs. AYA ML-DS (solid). Statistical analyses not conducted due to inadequate sample size.
